# Noncontrast Computed Tomography Markers Associated with Hematoma Expansion: Analysis of a Multicenter Retrospective Study

**DOI:** 10.3390/brainsci13040608

**Published:** 2023-04-03

**Authors:** Lianghong Yu, Mingpei Zhao, Yuanxiang Lin, Jiateng Zeng, Qiu He, Yan Zheng, Ke Ma, Fuxin Lin, Dezhi Kang

**Affiliations:** 1 Department of Neurosurgery, Neurosurgery Research Institute, The First Affiliated Hospital, Fujian Medical University, Fuzhou 350005, China; 2 Department of Neurosurgery, Binhai Branch of National Regional Medical Center, The First Affiliated Hospital, Fujian Medical University, Fuzhou 350005, China; 3 Fujian Institute for Brain Disorders and Brain Science, The First Affiliated Hospital, Fujian Medical University, Fuzhou 350005, China; 4 Fujian Provincial Clinical Research Center for Neurological Diseases, The First Affiliated Hospital, Fujian Medical University, Fuzhou 350005, China; 5 Clinical Research and Translation Center, The First Affiliated Hospital, Fujian Medical University, Fuzhou 350005, China

**Keywords:** intracerebral hemorrhage, hematoma expansion, hematoma heterogeneity, density categories of heterogeneous, non-contrast computed tomography, stroke

## Abstract

Background: Hematoma expansion (HE) is a significant predictor of poor outcomes in patients with intracerebral hemorrhage (ICH). Non-contrast computed tomography (NCCT) markers in ICH are promising predictors of HE. We aimed to determine the association of the NCCT markers with HE by using different temporal HE definitions. Methods: We utilized Risa-MIS-ICH trial data (risk stratification and minimally invasive surgery in acute intracerebral hemorrhage). We defined four HE types based on the time to baseline CT (BCT) and the time to follow-up CT (FCT). Hematoma volume was measured by software with a semi-automatic edge detection tool. HE was defined as a follow-up CT hematoma volume increase of >6 mL or a 33% hematoma volume increase relative to the baseline CT. Multivariable regression analyses were used to determine the HE parameters. The prediction potential of indicators for HE was evaluated using receiver-operating characteristic analysis. Results: The study enrolled 158 patients in total. The time to baseline CT was independently associated with HE in one type (odds ratio (OR) 0.234, 95% confidence interval (CI) 0.077–0.712, *p* = 0.011), and the blend sign was independently associated with HE in two types (OR, 6.203–6.985, both *p* < 0.05). Heterogeneous density was independently associated with HE in all types (OR, 6.465–88.445, all *p* < 0.05) and was the optimal type for prediction, with an area under the curve of 0.674 (*p* = 0.004), a sensitivity of 38.9%, and specificity of 96.0%. Conclusion: In specific subtypes, the time to baseline CT, blend sign, and heterogeneous density were independently associated with HE. The association between NCCT markers and HE is influenced by the temporal definition of HE. Heterogeneous density is a stable and robust predictor of HE in different subtypes of hematoma expansion.

## 1. Introduction

Acute spontaneous cerebral hemorrhage is the most severe and incurable type of stroke, with one-third of patients dying within the first month [1]. Hematoma expansion (HE) is a strong predictor of poor outcomes in patients with intracerebral hemorrhage (ICH) [2,3,4]. Markers that are predictive of HE can help in selecting patients for hemostatic treatment [5]. Therefore, the early identification of patients at high risk of HE is critical. The spot sign on computed tomographic angiography (CTA) has been shown to have a high predictive value for increased hematoma [5,6,7,8,9].

Furthermore, leakage and iodine signs are also sensitive predictors of HE [10,11]. However, in some countries and regions, the low popularity rate of CTA limits the use of these markers. In contrast to CTA, non-contrast computed tomography (NCCT) markers have the potential to be a low-cost and widely used tool for cerebral hemorrhage risk stratification [12]. NCCT markers can be classified as density and shape markers [13]. Swirl signs, heterogeneous density, black hole signs, blend signs, and blood-fluid levels are widely used as qualitative hematoma heterogeneity markers to predict HE [14,15,16,17,18]. Currently, there is no standardized definition of HE, and the relationship between predictors and HE varies according to different volume growth definitions of HE [16,19,20,21]. However, there is a lack of studies examining the relationship between predictors and HE under different temporal definitions. Consequently, we sought to determine the associations between NCCT markers and hematoma expansion under various temporal definitions and to identify stable markers for predicting hematoma expansion.

## 2. Materials and Methods

### 2.1. Study Design and Subjects

Our study is based on the Risa-MIS-ICH trial retrospective study database (Clinical Trials Identifier: NCT03862729, https://www.clinicaltrials.gov, accessed on 1 December 2021). As of November 2021, the retrospective study had enrolled 1040 patients with spontaneous supratentorial cerebral hemorrhage and 225 patients with time to baseline CT within 12 h and a follow-up CT within 72 h. The time to baseline CT (BCT) was defined as the time from symptom onset to CT scan immediately after admission to the hospital. The exclusion criteria were as follows: (1) the CT was not NCCT at baseline; (2) the surgical intervention was conducted before the follow-up CT; and (3) the CT image quality was not optimal. The Ethics Committee at Fujian Medical University’s First Affiliated Hospital approved the study (Ethical Approval Number: MRCTA, ECFAH of FMU [2018] 082-1).

### 2.2. Treatment

The patients in this study were treated according to the following two guidelines: Guidelines for the Management of Spontaneous Intracerebral Hemorrhage: A Guideline for Healthcare Professionals From the American Heart Association/American Stroke Association (2015) [22] and Chinese multidisciplinary expert consensus: Diagnosis and Treatment of Spontaneous Cerebral Hemorrhage (2015).

### 2.3. Imaging Evaluation

Admission and follow-up NCCT scans were obtained according to the standard clinical protocols with axial 5-mm section thickness. The swirl sign, black hole sign, blend sign, blood-fluid level, and ultraearly hematoma growth (uHG) are defined based on the previous literature [3,13,14,15,16,17,18]. Heterogeneous density is defined as at least three foci of hypoattenuation compared to the surrounding hematoma, as assessed on the axial NCCT slice with the most ICH. (Barras density scale = III, IV, or V) [16]. Supplementary Figure S1 shows images of several representative NCCT markers. Brainlab iPlan CMF 3.0 software (Brainlab, Heimstetten, Germany) assessed the volume of the hematoma using semi-automatic edge detection technology. Two experienced neurosurgeons independently reviewed all images and evaluated the presence or absence of various NCCT markers based strictly on the definitions. They were unaware of clinical information about the patient before the imaging evaluation. A reviewer was added to the panel in case of discrepancies, and the three resolved the issue by consensus. According to commonly used growth criteria, hematoma expansion was defined as an absolute growth volume of more than 6 mL or a relative growth of more than 33% [5,23,24,25]. Four types of hematoma growth were defined according to BCT < 6 or 12 h and FCT < 24 or 72 h.

### 2.4. Statistical Analysis

SPSS version 27.0 (SPSS Inc., Chicago, IL, USA) and MedCalc software (MedCalc 20.0, MedCalc, Mariakerke, Belgium) were used for the analysis. A two-tailed Student’s t-test was used to compare the continuous variables with a normal distribution reported as the mean (SD). Continuous variables with non-normal distributions were represented by the median (25th–75th quartile) and analyzed using the Mann–Whitney U test. Categorical variables were expressed as numbers (%), and distribution differences between groups were determined using the chi-square test or Fisher’s test. We used univariate analysis to find potential essential factors that could influence HE. Elements with *p* < 0.05 in the univariate analysis were forced into the multiple regression model, adjusting for potential predictors of *p* < 0.05 in other types. Receiver-operating characteristic curves were drawn to assess independent predictors for predicting HE. The inter-rater and intra-rater reliability was calculated through the intraclass correlation coefficient (ICC) in the measurement of hematoma volume. For an ICC, moderate agreement was considered as 0.41 to 0.60, substantial agreement as 0.61 to 0.80, and excellent as 0.81 to 1.00. All tests were 2-tailed, and *p* < 0.05 was considered statistically significant.

## 3. Results

### 3.1. Characteristics of Study Patients

A total of 158 ICH patients were included in this study and were divided into four types according to different CT time definitions (Supplementary Figure S2). HE was observed in 19.1% to 21.2% of ICH patients. The average age of the patients was 61.0 ± 12.5 years; 81.0% were male, 69.6% suffered from hypertension, and 18.3% suffered from diabetes mellitus. At admission, the NCCT scan revealed that 82.3% of patients had deep ICH, 29.7% had combined IVH, and 12.0% had combined hydrocephalus. The two raters’ ICH volumes at the baseline CT and the follow-up CT were the same (inter-rater ICC [95% CI]:0.99 [0.99–1.00]). Moreover, there was no difference between intra-rater assessments (intra-rater ICC [95% CI]:0.99 [0.99–1.00]). Detailed clinical and imaging information for the patients is shown in Table 1.

### 3.2. Association between NCCT Markers and Hematoma Expansion for Different Definitions

In the definition type of BCT within 6 h and FCT within 24 h, patients with HE were more likely to present as heterogeneous (*p* < 0.001) and presented with a shorter time to baseline CT (*p* = 0.005) and a larger uHG (*p* = 0.022) than those patients without HE (Table 2). The univariate analysis demonstrated that “heterogeneous” (*p* < 0.001, OR (95% CI), 15.061 (3.380–67.106)), time to baseline CT (*p* = 0.006, OR (95% CI), 0.504 (0.309–0.821)), and uHG (*p* = 0.017, OR (95% CI), 1.048 (1.008–1.090)) were associated with HE (Table 3). Multivariable analyses showed that “heterogeneous” (*p* < 0.001, 40.536 (5.366–306.223)) and the time to baseline CT (*p* = 0.009, 0.278 (0.106–0.729)) were the independent risk factors, and the association remained significant after adjustment (Table 4). In the definition type of BCT within 6 h and FCT within 72 h, patients with HE were more likely to present as “heterogeneous” (*p* < 0.001), blend sign (*p* = 0.001), and presented with a shorter time to baseline CT (*p* = 0.023) and a larger uHG (*p* = 0.047) than those patients without HE (Table 2). The univariate analysis demonstrated that heterogeneous (*p* < 0.001, OR (95% CI), 10.471 (2.832–38.711)), blend sign (*p* = 0.001, OR (95% CI), 8.653 (2.291–32.677)), time to baseline CT (*p* = 0.025, OR (95% CI), 0.665 (0.465–0.949)), uHG (*p* = 0.016, OR (95% CI), and 1.044 (1.008–1.081)) were associated with HE (Table 3). Multivariable analyses showed that heterogeneous (*p* = 0.002, 8.833 (2.165–36.037)) and blend sign (*p* = 0.008, 7.121 (1.651–30.709)) were the independent risk factors, and the association remained significant after adjustment (Table 4). In the definition type of BCT within 12 h and FCT within 24 h, patients with HE were more likely to show “heterogeneous” (*p* < 0.001) and a black hole sign (*p* = 0.030), and to have a shorter time to baseline CT (*p* = 0.030) and a larger uHG (*p* = 0.010) than patients without HE (Table 5). The univariate analysis demonstrated that heterogeneous (*p* < 0.001, OR (95% CI), 12.714 (3.376–47.878)), black hole sign (*p* = 0.016, OR (95% CI), 5.176 (1.350–19.847)), uHG (*p* = 0.012, OR (95% CI), and 1.047 (1.010–1.086)) were associated with HE (Table 6). Multivariable analyses showed that heterogeneous (*p* = 0.008, 11.259 (1.873–67.687)) and uHG (*p* = 0.036, 1.044 (1.003–1.087)) were the independent risk factors, but only heterogeneous remains significant after adjustment. (Table 4). In the definition type of BCT within 12 h and FCT within 72 h, patients with HE were more likely to have diabetes mellitus (*p* < 0.001), “heterogeneous” (*p* < 0.001), a black hole sign (*p* = 0.024), and a blend sign (*p* < 0.001) were less likely to have intraventricular hemorrhage (IVH) (*p* = 0.022), and presented with a shorter time to baseline CT (*p* = 0.018) and a larger uHG (*p* = 0.022) than those patients without HE (Table 5). The univariate analysis demonstrated that diabetes mellitus (*p* = 0.045, OR (95% CI), 3.147 (1.027–9.639)), IVH (*p* = 0.029, OR (95% CI), 0.289 (0.095–0.880)), heterogeneous (*p* < 0.001, OR (95% CI), 9.603 (3.155–29.231)), black hole sign (*p* = 0.045, OR (95% CI), 3.147 (1.027–9.639)), blend sign (*p* < 0.001, OR (95% CI), 10.696 (2.973–38.474)), uHG (*p* = 0.007, OR (95% CI), and 1.045 (1.012–1.079)) were associated with HE (Table 6). Multivariable analyses showed that heterogeneous (*p* = 0.009, 6.473 (1.579–26.541)) and uHG (*p* = 0.015, 6.197 (1.428–26.886)) were the independent risk factors, and the association remained significant after adjustment (Table 4).

### 3.3. Independent Predictive Value of Significant Predictors for HE

**Figure 1:**
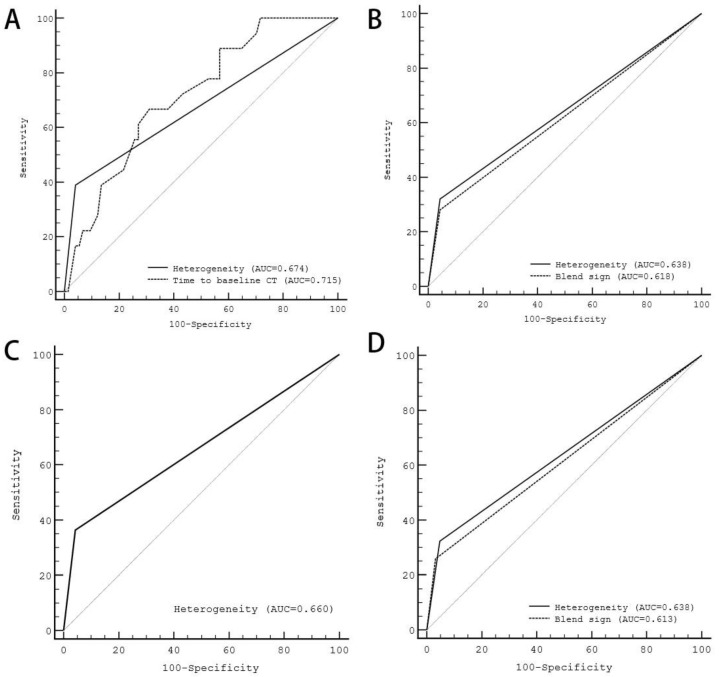
Receiver-operating characteristic curve analysis of significant predictors and different hematoma expansion definitions. (**A**) The AUC of “heterogeneous” was 0.674 (*p* = 0.004), and the time to baseline CT was 0.715 (*p* = 0.001) in the definition type of BCT within 6 h and FCT within 24 h. (**B**) The AUC of “heterogeneous” was 0.638 (*p* = 0.005) and the blend sign was 0.618 (*p* = 0.012) in the definition type of BCT within 6 h and FCT within 72 h. (**C**) The AUC of “heterogeneous” was 0.660 (*p* = 0.003) in the definition type of BCT within 12 h and FCT within 24 h. (**D**) The AUC of “heterogeneous” was 0.638 (*p* = 0.002) and the blend sign was 0.613 (*p* = 0.005) in the definition type of BCT within 12 h and FCT within 72 h. AUC, the area under the curve; BCT, time to baseline CT; FCT, time to follow-up CT.

The receiver-operating characteristic (ROC) curve analysis revealed that the area under the curve (AUC) of the heterogeneous type was 0.674 (*p* = 0.004) and the time to baseline CT was 0.715 (*p* = 0.001) for the type with BCT within 6 h and FCT within 24 h (Figure 1). The heterogeneity was 0.638 (*p* = 0.005) and the blend sign was 0.618 (*p* = 0.012) in the type with BCT within 6 h and FCT within 72 h (Figure 1). The heterogeneity was 0.660 (*p* = 0.003) in the type with BCT within 12 h and FCT within 24 h; meanwhile, the heterogeneity was 0.638 (*p* = 0.002) and the blend sign was 0.613 (*p* = 0.005) in the type with BCT within 12 and FCT within 72 h (Figure 1). “Heterogeneous” demonstrates consistently sound predictive value for any definition, predicting HE with sensitivity, specificity, positive predictive value, negative predictive value, and area under the curve, as detailed in Table 7.

## 4. Discussion

Our study showed that heterogeneous hematoma was independently associated with the risk of hematoma expansion. However, the association of other predictors with hematoma expansion varies depending on each definition of HE. The blend sign was independently associated with HE in half of the descriptions, while time to baseline CT was independently associated with HE in only 25% of the definitions. 

No standard definition of hematoma expansion is currently available. Reports on hematoma expansion refer to the increase in volume and the corresponding time. In earlier studies, hematoma expansion was defined as an absolute increase of 20 mL or a relative increase of >40% [26], but this definition is now rarely used. Later, Brott et al. defined hematoma expansion as an increase in the relative volume of greater than 33% [27,28]; this definition is still used today [29,30]. Currently, hematoma expansion is mainly defined by a combination of relative and absolute growth, with >33% or >6 mL [3,9,31,32,33,34,35] and >33% or >12.5 mL [17,36,37] being the standard; in particular, >33% or >6 mL is widely used. Different definitions of volume growth affect the relationship between predictors and HE [16,19,20,21]. The time aspect of hematoma definitions is often ignored. Due to the relative instability of early hematomas, hematoma growth is more common among patients with early ICH. The activation of the coagulation system allows the hematoma to become progressively more stable as time passes. Consequently, a CT scan performed late after the onset of the disease may be less likely to detect an enlarged hematoma, and it is more accurate to say that the hematoma has grown to a stable state than that it has not grown. H. Bart Brouwers et al. confirmed that a shorter time to computed tomography (≤6 vs. >6 h) independently predicted HE [31], and patients with a baseline CT time < 6 h were included in a subsequent study of hematoma expansion [4,20,35]. Considering that some patients cannot reach the hospital and receive a CT scan in time, especially in remote areas, and 17% of hematoma expansion occurred 6 h after onset [38], we consider it reasonable to include baseline CT time < 12 h in the study. In addition, there is no uniformity in the definition of follow-up CT time, but it is generally within 72 h [35,39,40]. As a result, when studying the relationship between predictors and HE, the type of HE definition used should be noted.

Contrast extravasation observed on CTA is a significant indicator for identifying ongoing bleeding [5]. The spot sign is a strong predictor of HE [5]; this sign is characterized as one or more 1 to 2 mm foci of enhancement within the hematoma on CTA source pictures [6], and the enhancement foci are generally considered to be manifestations of the extravasation of the contrast medium from the blood vessels. Later, the leakage and iodine signs on CTA images were also considered risk factors for HE sensitivity [10,11]. CTA is an ideal screening technique for cerebral hemorrhage because it can detect the marker that predicts hematoma expansion and identify bleeding caused by vascular malformation. However, CTA tests are not readily accessible, limiting their extensive application in clinical practice.

In contrast to CTA, NCCT is readily available in most stroke hospitals. NCCT markers can be divided into two types based on shape and density [13,24], and NCCT markers representing hematoma density are more common than those representing shapes. NCCT markers based on hematoma density, such as the swirl sign, black hole sign, blend sign, blood-fluid level, and density category of hematoma, were described as “heterogeneous”, representing local or global hematoma heterogeneity. Notably, the definition of NCCT markers is relatively complex; there is no consensus, and there is inter-reviewer variability [41]. Recent studies have shown that quantitative NCCT markers can be used to predict hematoma expansion [42,43]; however, the need for specialist software may limit the widespread use of this technique. In addition, the artificial intelligence model offers improved specificity and sensitivity in predicting HE [44]. In conclusion, predicting HE using NCCT images is a promising prospect. We recommend that all NCCT markers be prospectively validated and evaluated for inter- and intra-rater reliability.

Fisher et al. proposed an "avalanche" model to explain the mechanism of hematoma expansion [45]. In this model, the hematoma stretches the surrounding brain tissue, causing numerous small blood vessels to be cut and ruptured. As the hematoma increases further, the pressure on the surrounding brain tissue increases, and the hemorrhage stops when the hematoma and surrounding tissue pressure are balanced [16,46]. In this model, the earlier hematoma continuously adds new liquid blood until the bleeding stops. The clot shrinks after bleeding to obtain hemostasis, the serum is extruded, and the CT attenuation of the hematoma increases [47]. Fluid blood from an active hemorrhage hypoattenuates on a CT scan relative to the surrounding brain or high attenuation thrombus [46]. Thus, hematoma heterogeneity may represent the coexistence of relatively early high-attenuation hematomas with relatively new bleeding low-attenuation hematomas in active bleeding. We speculate that, if the hematoma is slowly and continuously expanding, the further active bleeding may be of multiple origins, which may be more consistent with a heterogeneous density score. This type of hematoma expansion is probably the most common. However, if there is active bleeding on top of a relatively stable hematoma, i.e., if there are two peaks of bleeding, this may be more consistent with blend sign.

This study has several limitations. First, the sample size was relatively small. Second, the study was retrospective and had all the typical constraints of a retrospective analysis. Third, all of the definitions utilized herein commonly used growth volume (>6 mL or 33%), which may limit the applicability of our results. Fourth, patients with more than 12 h between onset and the initial CT scan were excluded from this study; therefore, our results may not apply to this population. Fifth, this was an NCCT-based study that did not compare NCCT markers to the CTA spot sign. Finally, this study only showed the association of NCCT with HE and offered no further investigation of the association of NCCT with poor outcomes, limiting the clinical value of the results.

In summary, our study showed that the time to baseline CT, blend sign, and heterogeneous density was independently associated with HE in certain subtypes. We found that different temporal HE definitions affected the relationship between predictors and HE, but heterogeneous hematoma independently predicted HE in all definition types. Heterogeneous hematoma as a stable independent predictor of HE may be a substitute when CTA scans cannot be performed, and individuals with heterogeneous hematoma may be candidates for therapy that limits early hematoma expansion. In the subsequent research, our results will be validated using prospective, multicenter, large-sample-size RIS-MIS-ICH project data.

## Figures and Tables

**Table 1:** Clinical and imaging characteristics of patients with intracerebral hemorrhage who were included in the study.

Variable	Total (*n* = 158)
Age, y, mean (SD)	61.0 (12.5)
Male sex, n (%)	128 (81.0)
Hypertension, n (%)	110 (69.6)
Diabetes mellitus, n (%)	29 (18.3)
Oral anticoagulants, n (%)	3 (1.9%)
Oral antiplatelet drugs, n (%)	4 (2.5%)
Admission SBP, mmHg, mean (SD)	163.6 (27.7)
Admission DBP, mmHg, mean (SD)	95.4 (16.4)
Baseline GCS, median (IQR)	13 (10–15)
Deep ICH, n (%)	130 (82.3)
IVH, n (%)	47 (29.7)
Hydrocephalus, n (%)	19 (12.0)
Density category	
Homogeneous, n (%)	142 (89.9)
Heterogeneous, n (%)	16 (10.1)
Swirl sign, n (%)	38 (24.1)
Black hole sign, n (%)	15 (9.5)
Blend sign, n (%)	12 (7.6)
Blood-fluid level, n (%)	4 (2.5)
Time to baseline CT, h, median (IQR)	3.5 (2.0–6.3)
ICH volume, mL, median (IQR)	24.3 (14.5–36.5)
UHG, mL/h, median (IQR)	5.5 (3.3–13.6)
WBC, 10^9^/L, mean (SD)	9.5 (3.3)
HGB, g/L, mean (SD)	142.3 (18.0)
PLT, 10^9^/L, mean (SD)	212.6 (63.7)
GLU, mmol/L, mean (SD)	7.4 (3.4)

SD, standard deviation; SBP, systolic blood pressure; DBP, diastolic blood pressure; GCS, Glasgow Coma Scale; IQR, interquartile range; ICH, intracerebral hemorrhage; IVH, intraventricular hemorrhage; CT, computed tomography; UHG, ultraearly hematoma growth; WBC, white blood cell; HGB, hemoglobin; PLT, platelet; GLU, blood glucose.

**Table 2:** Comparison of baseline clinical and radiological characteristics of individuals with and without hematoma expansion for the time to baseline CT within 6 h.

Variable	Hematoma Expansion for BCT within 6 h and FCT within 24 h (*n* = 92)			Hematoma Expansion for BCT within 6 h and FCT within 72 h (*n* = 118)		
	Yes (*n* = 18)	No (*n* = 74)	*p*-Value	Yes (*n* = 25)	No (*n* = 93)	*p*-Value
Age, y, mean (SD)	59.8 (13.3)	61.0 (12.3)	0.733	57.4 (12.6)	60.9 (12.0)	0.206
Male Sex, n (%)	16 (88.9)	57 (77.0)	0.429	22 (88.0)	74 (79.6)	0.502
Hypertension, n (%)	12 (66.7)	56 (75.7)	0.630	15 (60.0)	68 (73.1)	0.202
Diabetes mellitus, n (%)	3 (16.7)	6 (8.1)	0.513	5 (20.0)	7 (7.5)	0.145
Admission SBP, mmHg, mean (SD)	168.9 (30.7)	167.7 (26.2)	0.856	163.8 (29.8)	163.8 (26.2)	0.998
Admission DBP, mmHg, mean (SD)	97.1 (17.8)	98.6 (15.7)	0.725	91.2 (16.7)	98.1 (16.1)	0.063
Baseline GCS, median (IQR)	13 (9–15)	13 (10–15)	0.960	14 (9.5–15)	13 (10–15)	0.699
Deep ICH, n (%)	16 (88.9)	62 (83.8)	0.861	23 (92.0)	79 (84.9)	0.558
IVH, n (%)	3 (16.7)	23 (31.1)	0.223	5 (20.0)	24 (25.8)	0.549
Hydrocephalus, n (%)	2 (11.1)	9 (12.2)	0.902	1 (4.0)	12 (12.9)	0.367
Heterogeneity, n (%)	7 (38.9)	3 (4.1)	<0.001	8 (32.0)	4 (4.3)	<0.001
Swirl sign, n (%)	6 (33.3)	16 (21.6)	0.461	7 (28.0)	24 (25.8)	0.825
Black hole sign, n (%)	3 (16.7)	4 (5.4)	0.262	3 (12.0)	8 (8.6)	0.896
Blend sign, n (%)	3 (16.7)	4 (5.4)	0.262	7 (28.0)	4 (4.3)	0.001
Blood-fluid level, n (%)	1 (5.6)	2 (2.7)	0.484	1 (4.0)	3 (3.2)	0.849
Time to baseline CT, h, median (IQR)	1.8 (1.3–2.9)	2.8 (1.8–4.1)	0.005	2.0 (1.6–3.4)	2.8 (1.9–4.1)	0.023
ICH volume, mL, median (IQR)	24.9 (16.9–36.0)	21.0 (12.9–31.3)	0.340	24.3 (15.9–33.6)	21.6(13.5–31.5)	0.453
UHG, mL/h, median (IQR)	14.2 (6.8–30.3)	7.2 (3.8–14.9)	0.022	10.8 (5.1–26.3)	7.2 (4.0–15.5)	0.047
WBC, 10^9^/L, mean (SD)	9.6 (4.8)	9.5 (2.9)	0.869	8.8 (4.6)	9.6 (3.0)	0.320
HGB, g/L, mean (SD)	142.6 (18.8)	140.4 (19.0)	0.665	143.9 (17.4)	141.2 (18.2)	0.497
PLT, 10^9^/L, mean (SD)	208.6 (77.3)	223.4 (57.8)	0.367	199.4 (74.3)	216.5 (61.4)	0.242
GLU, mmol/L, mean (SD)	8.5 (4.5)	7.6 (3.4)	0.394	8.3 (4.5)	7.4 (3.2)	0.244

BCT, time to baseline CT; FCT, time to follow-up CT; SD, standard deviation; SBP, systolic blood pressure; DBP, diastolic blood pressure; GCS, Glasgow Coma Scale; IQR, interquartile range; ICH, intracerebral hemorrhage; IVH, intraventricular hemorrhage; CT, computed tomography; UHG, ultraearly hematoma growth; WBC, white blood cell; HGB, hemoglobin; PLT, platelet; GLU, blood glucose.

**Table 3:** Univariate analysis of the predictors of hematoma expansion for the time to baseline CT within 6 h.

Variable	BCT within 6 h and FCT within 24 h (*n* = 92)		BCT within 6 h and FCT within 72 h (*n* = 118)	
	OR (95% CI)	*p*-Value	OR (95% CI)	*p*-Value
Age	0.993 (0.952–1.035)	0.730	0.975 (0.939–1.014)	0.206
Sex	0.419 (0.087–2.008)	0.277	0.531 (0.144–1.963)	0.343
Hypertension	0.643 (0.211–1.960)	0.437	0.551 (0.219–1.387)	0.206
Diabetes mellitus	2.267 (0.509–10.102)	0.283	3.071 (0.883–10.683)	0.078
Admission SBP	1.002 (0.983–1.021)	0.854	1.000 (0.984–1.017)	0.998
Admission DBP	0.994 (0.962–1.027)	0.722	0.973 (0.945–1.002)	0.067
Baseline GCS	0.961 (0.812–1.136)	0.641	0.997 (0.860–1.157)	0.973
Deep ICH	1.548 (0.314–7.628)	0.591	2.038 (0.431–9.627)	0.369
IVH	0.443 (0.117–1.683)	0.232	0.719 (0.243–2.126)	0.551
Hydrocephalus	0.903 (0.177–4.593)	0.902	0.281 (0.035–2.274)	0.234
Heterogeneity	15.061 (3.380–67.106)	<0.001	10.471 (2.832–38.711)	<0.001
Swirl sign	1.812 (0.588–5.586)	0.300	1.118 (0.416–3.006)	0.825
Black hole sign	3.500 (0.708–17.291)	0.124	1.449 (0.355–5.918)	0.606
Blend sign	3.500 (0.708–17.291)	0.124	8.653 (2.291–32.677)	0.001
Blood-fluid level	2.118 (0.181–24.736)	0.550	1.250 (0.124–12.562)	0.850
Time to baseline CT	0.504 (0.309–0.821)	0.006	0.665 (0.465–0.949)	0.025
ICH volume	1.015 (0.985–1.045)	0.337	1.011 (0.983–1.040)	0.442
UHG	1.048 (1.008–1.090)	0.017	1.044 (1.008–1.081)	0.016
WBC	1.013 (0.870–1.180)	0.867	0.930 (0.805–1.073)	0.318
HGB	1.006 (0.978–1.035)	0.661	1.009 (0.983–1.035)	0.493
PLT	0.996 (0.987–1.005)	0.363	0.996 (0.989–1.003)	0.241
GLU	1.058 (0.930–1.203)	0.394	1.069 (0.954–1.198)	0.248

BCT, time to baseline CT; FCT, time to follow-up CT; OR, odds ratio; 95% CI, 95% confidence interval; SBP, systolic blood pressure; DBP, diastolic blood pressure; GCS, Glasgow Coma Scale; ICH, intracerebral hemorrhage; IVH, intraventricular hemorrhage; CT, computed tomography; UHG, ultraearly hematoma growth; WBC, white blood cell; HGB, hemoglobin; PLT, platelet; GLU, blood glucose.

**Table 4:** Multivariate analysis of the predictors for hematoma expansion in different types.

	Unadjusted		Adjusted *	
	OR (95% CI)	*p*-Value	OR (95% CI)	*p*-Value
BCT within 6 h and FCT within 24 h				
Heterogeneity	40.536 (5.366–306.223)	<0.001	88.445(5.387–1452.191)	0.002
Time to baseline CT	0.278 (0.106–0.729)	0.009	0.234 (0.077–0.712)	0.011
UHG	0.971 (0.910–1.036)	0.368	0.973 (0.909–1.042)	0.437
BCT within 6 h and FCT within 72 h				
Heterogeneity	8.833 (2.165–36.037)	0.002	31.703 (3.036–331.026)	0.004
Blend sign	7.121 (1.651–30.709)	0.008	6.985 (1.364–35.776)	0.020
Time to baseline CT	0.722 (0.414–1.259)	0.251	0.660 (0.360–1.209)	0.179
UHG	1.011 (0.956–1.071)	0.695	1.024 (0.963–1.088)	0.447
BCT within 12 h and FCT within 24 h				
Heterogeneity	11.259 (1.873–67.687)	0.008	10.478 (1.657–66.252)	0.013
Black hole sign	1.061 (0.136–8.293)	0.955	1.214 (0.161–9.173)	0.851
UHG	1.044 (1.003–1.087)	0.036	1.044 (0.990–1.100)	0.110
BCT within 12 h and FCT within 72 h				
Diabetes mellitus	3.255 (0.838–12.645)	0.088	3.259 (0.836–12.702)	0.089
IVH	0.368 (0.108–1.261)	0.112	0.367 (0.105–1.281)	0.116
Heterogeneity	6.473 (1.579–26.541)	0.009	6.465 (1.569–26.637)	0.010
Black hole sign	1.292 (0.296–5.634)	0.733	1.292 (0.296–5.635)	0.733
Blend sign	6.197 (1.428–26.886)	0.015	6.203 (1.424–27.014)	0.015
UHG	1.044 (1.004–1.086)	0.029	1.045 (0.995–1.097)	0.081

OR, odds ratio; 95% CI, 95% confidence interval; BCT, time to baseline CT; FCT, time to follow-up CT; CT, computed tomography; UHG, ultraearly hematoma growth; IVH, intraventricular hemorrhage. * Adjusting for potential predictors of *p* < 0.05 in other types.

**Table 5:** Comparison of baseline clinical and radiological characteristics of individuals with and without hematoma expansion for time to baseline CT within 12 h.

Variable	Hematoma Expansion for BCT within 12 h and FCT within 24 h (*n* = 115)			Hematoma Expansion for BCT within 12 h and FCT within 72 h (*n* = 158)		
	Yes (*n* = 22)	No (*n* = 93)	*p*-Value	Yes (*n* = 31)	No (*n* = 127)	*p*-Value
Age, y, mean (SD)	59.6 (12.6)	60.8 (12.2)	0.669	57.7 (12.5)	61.8 (12.4)	0.106
Male Sex, n (%)	20 (90.9)	71 (76.3)	0.222	28 (90.3)	100 (78.7)	0.140
Hypertension, n (%)	16 (72.7)	66 (71.0)	0.870	20 (64.5)	90 (70.9)	0.491
Diabetes mellitus, n (%)	3 (13.6)	7 (7.5)	0.621	20 (64.5)	9 (7.1)	<0.001
Admission SBP, mmHg, mean (SD)	171.4 (31.8)	165.2 (27.1)	0.354	165.1 (30.5)	163.3 (27.1)	0.747
Admission DBP, mmHg, mean (SD)	97.2 (20.3)	96.4 (15.6)	0.849	97.0 (19.0)	95.0 (15.7)	0.552
Baseline GCS, median (IQR)	13 (9.75–15)	13 (10–15)	0.878	14 (15–10)	13 (15–10)	0.542
Deep ICH, n (%)	19 (86.4)	75 (80.6)	0.751	28 (90.3%)	102 (80.3%)	0.191
IVH, n (%)	4 (18.2)	33 (35.5)	0.118	4 (12.9)	43 (33.9)	0.022
Hydrocephalus, n (%)	3 (13.6)	13 (14.0)	0.967	3 (9.7)	16 (12.6)	0.888
Heterogeneity, n (%)	8 (36.4)	4 (4.3)	<0.001	10 (32.3)	6 (4.7)	<0.001
Swirl sign, n (%)	7 (31.8)	20 (21.5)	0.305	8 (25.8)	30 (23.6)	0.799
Black hole sign, n (%)	5 (22.7)	5 (5.4)	0.030	6 (19.3)	9 (7.1)	0.024
Blend sign, n (%)	3 (13.6)	4 (4.3)	0.250	8 (25.8)	4 (3.1)	<0.001
Blood–fluid level, n (%)	1 (4.5)	2 (2.2)	0.474	2 (6.5)	2 (1.6)	0.173
Time to baseline CT, h, median (IQR)	2.1 (1.6–3.7)	3.5 (2.2–5.5)	0.030	2.1 (4.5–1.6)	3.7 (6.5–2.3)	0.018
ICH volume, mL, median (IQR)	27.4 (19.1–44.4)	23.3 (13.7–36.8)	0.146	26.2 (40.0–17.8)	23.8 (36.4–14.1)	0.278
UHG, mL/h, median (IQR)	11.2 (4.8–25.5)	5.6 (3.3–12.8)	0.010	8.8 (4.2–24.8)	5.2 (3.3–11.7)	0.022
WBC, 10^9^/L, mean (SD)	9.1 (4.7)	9.5 (2.9)	0.619	8.7 (4.4)	9.7 (3.0)	0.142
HGB, g/L, mean (SD)	138.7 (21.3)	141.6 (18.0)	0.507	141.1 (19.4)	142.6 (17.7)	0.676
PLT, 10^9^/L, mean (SD)	201.6 (80.4)	219.7 (56.5)	0.219	200.1 (78.4)	215.6 (59.5)	0.223
GLU, mmol/L, mean (SD)	8.6 (4.9)	7.5 (3.2)	0.166	8.3 (4.8)	7.2 (2.9)	0.248

BCT, time to baseline CT; FCT, time to follow-up CT; SD, standard deviation; SBP, systolic blood pressure; DBP, diastolic blood pressure; GCS, Glasgow Coma Scale; IQR, interquartile range; ICH, intracerebral hemorrhage; IVH, intraventricular hemorrhage; CT, computed tomography; UHG, ultraearly hematoma growth; WBC, white blood cell; HGB, hemoglobin; PLT, platelet; GLU, blood glucose.

**Table 6:** Univariate analysis of the predictors of hematoma expansion for a time to baseline CT within 12 h.

Variable	BCT within 12 h and FCT within 24 h (*n* = 115)		BCT within 12 h and FCT within 72 h (*n* = 158)	
	OR (95% CI)	*p*-Value	OR (95% CI)	*p*-Value
Age	0.992 (0.954–1.031)	0.666	0.973 (0.941–1.006)	0.108
Sex	0.323 (0.070–1.491)	0.147	0.397 (0.112–1.405)	0.152
Hypertension	1.091 (0.386–3.085)	0.870	0.747 (0.326–1.713)	0.492
Diabetes mellitus	1.940 (0.459–8.195)	0.367	3.147 (1.027–9.639)	0.045
Admission SBP	1.008 (0.991–1.025)	0.352	1.002 (0.988–1.017)	0.745
Admission DBP	1.003 (0.975–1.031)	0.847	1.007 (0.984–1.032)	0.549
Baseline GCS	0.971 (0.827–1.141)	0.721	1.011 (0.879–1.164)	0.876
Deep ICH	1.520 (0.405–5.701)	0.535	2.288 (0.643–8.133)	0.201
IVH	0.404 (0.126–1.294)	0.127	0.289 (0.095–0.880)	0.029
Hydrocephalus	0.972 (0.252–3.753)	0.967	0.743 (0.202–2.730)	0.655
Heterogeneity	12.714 (3.376–47.878)	<0.001	9.603 (3.155–29.231)	<0.001
Swirl sign	1.703 (0.611–4.745)	0.308	1.125 (0.456–2.774)	0.799
Black hole sign	5.176 (1.350–19.847)	0.016	3.147 (1.027–9.639)	0.045
Blend sign	3.513 (0.726–17.001)	0.118	10.696 (2.973–38.474)	<0.001
Blood-fluid level	2.167 (0.188–25.030)	0.536	4.310 (0.583–31.886)	0.152
Time to baseline CT	0.855 (0.692–1.056)	0.146	0.878 (0.751–1.026)	0.101
ICH volume	1.022 (0.996–1.049)	0.102	1.016 (0.992–1.041)	0.199
UHG	1.047 (1.010–1.086)	0.012	1.045 (1.012–1.079)	0.007
WBC	0.963 (0.829–1.117)	0.616	0.906 (0.795–1.033)	0.142
HGB	0.992 (0.968–1.016)	0.504	0.995 (0.974–1.017)	0.674
PLT	0.995 (0.987–1.003)	0.218	0.996 (0.990–1.002)	0.223
GLU	1.081 (0.966–1.210)	0.174	1.082 (0.977–1.199)	0.129

BCT, time to baseline CT; FCT, Time to follow-up CT; OR, odds ratio; 95% CI, 95% confidence interval; SBP, systolic blood pressure; DBP, diastolic blood pressure; GCS, Glasgow Coma Scale; ICH, intracerebral hemorrhage; IVH, intraventricular hemorrhage; CT, computed tomography; UHG, ultraearly hematoma growth; WBC, white blood cell; HGB, hemoglobin; PLT, platelet; GLU, blood glucose.

**Table 7:** Sensitivity, specificity, positive predictive value, negative predictive value, and area under the curve of hematoma heterogeneity to predict hematoma expansion in different types.

Variable	Sensitivity (%)	Specificity (%)	PPV (%)	NPV (%)	AUC
BCT within 6 h and FCT within 24 h	38.9	96.0	70.0	86.6	0.674
BCT within 6 h and FCT within 72 h	32.0	95.7	66.7	84.0	0.638
BCT within 12 h and FCT within 24 h	36.4	95.7	66.7	86.4	0.660
BCT within 12 h and FCT within 72 h	32.3	95.3	62.5	85.2	0.638

BCT, time to baseline CT; FCT, time to follow-up CT; PPV, positive predictive value; NPV, negative predictive value; AUC, area under the curve; CT, computed tomography.

## Data Availability

The authors will make the raw data supporting the results of this article available.

## Supplementary Materials

The following supporting information can be downloaded at: https://www.mdpi.com/article/10.3390/brainsci13040608/s1, Supplementary Figure S1. Several instances of NCCT markers. Axial images of four different ICH patients. (A) Density cat-egory III (white arrow) is classified as “heterogeneous” while (B) density category I (white arrow) is characterized as “homogeneous”. Both (A) and (B) are the largest levels of the axial scan of the hematoma. (B) is defined as the black hole sign. The low-attenuation region (white arrow) is encased within the high-attenuation hematoma. The density difference between the two is approximately 36 Hounsfield units (HU). (C) means for blend sign. There is a visible demarcation (black dashed line) between the low attenuation area and the neighboring high attenuation area, and the density dif-ference between the two is around 18 HU. (D) is defined as swirl sign. The relatively low-attenuation region (white arrow) is wrapped within the high-attenuation hematoma. The border between the two is indistinct, with a density difference of 26 HU. Supplementary Figure S2. Flow chart of patients enrolled in the study. CT, computed tomography; NCCT, non-contrast computed tomography; BCT, time to baseline CT; FCT, time to follow-up CT.
