# Supplementary material for: Noncontrast Computed Tomography Markers Associated with Hematoma Expansion: Analysis of a Multicenter Retrospective Study
Source: Brain Sci. 2023 Apr 3;13(4):608. doi: 10.3390/brainsci13040608 (PMC10136660; doi:10.3390/brainsci13040608)
Supplement: Supplementary file 1 [file brainsci-13-00608-s001.zip › brainsci-2237010-supplementary.pdf]

Supplementary Materials

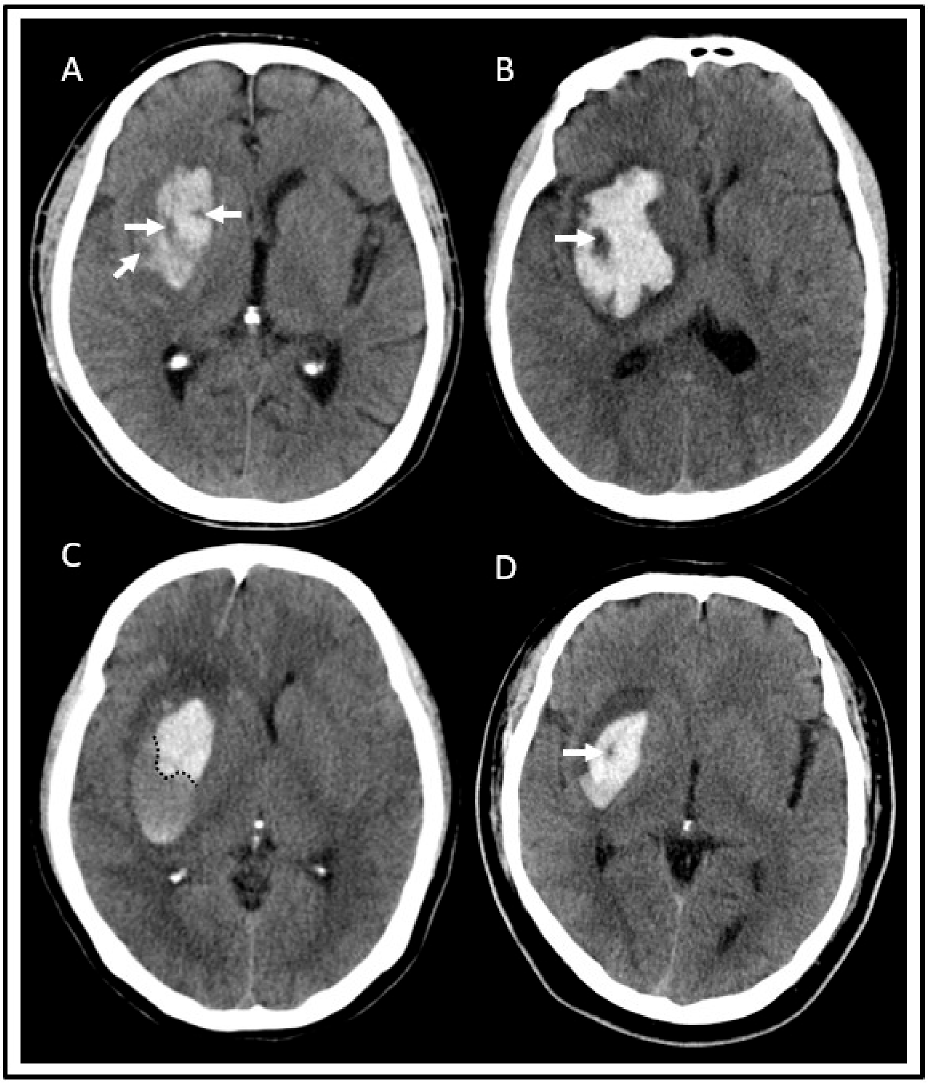

Figure S1. Several instances of NCCT markers. Axial images of four different ICH patients. (A) Density cat-egory III (white arrow) is classified as "heterogeneous" while (B) density category I (white arrow) is characterized as "homogeneous". Both (A) and (B) are the largest levels of the axial scan of the hematoma. (B) is defined as the black hole sign. The low-attenuation region (white arrow) is encased within the high-attenuation hematoma. The density difference between the two is approximately 36 Hounsfield units (HU). (C) means for blend sign. There is a visible demarcation (black dashed line) between the low attenuation area and the neighboring high attenuation area, and the density dif-ference between the two is around 18 HU. (D) is defined as swirl sign. The relatively low-attenuation region (white arrow) is wrapped within the high-attenuation hematoma. The border between the two is indistinct, with a density difference of 26 HU.

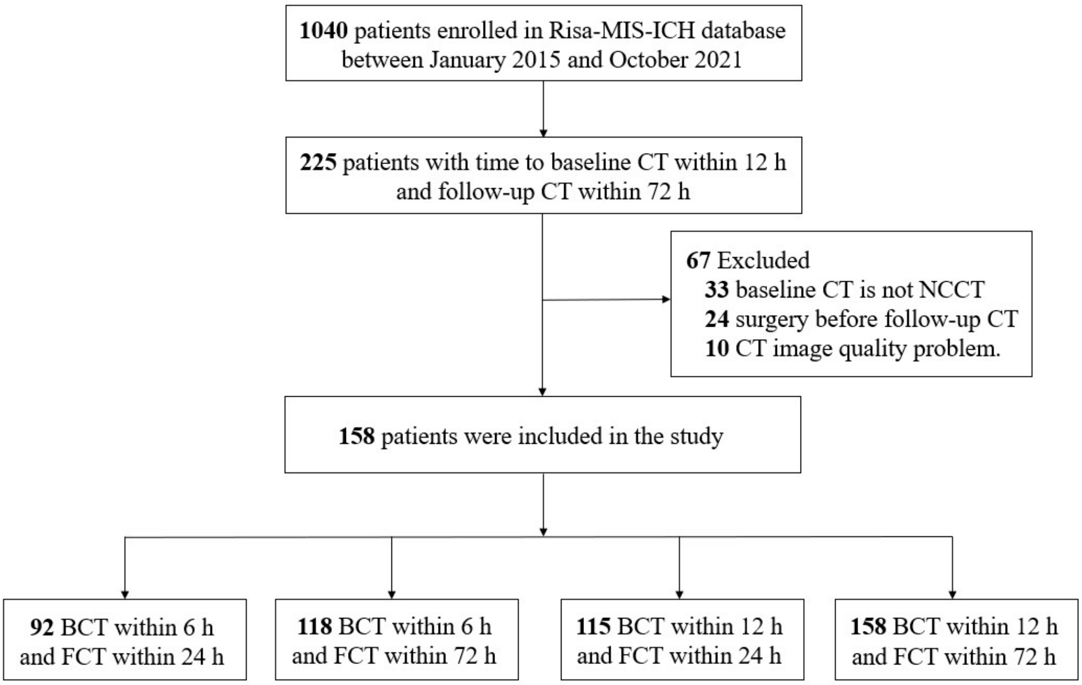

Figure S2. Flow chart of patients enrolled in the study.
CT, Computed tomography; NCCT, Non-contrast computed tomography; BCT, Time to baseline CT; FCT, Time to follow-up CT.
